# Regretful pleasure: Toward an understanding of flow cost in media use

**DOI:** 10.1371/journal.pone.0268194

**Published:** 2022-05-12

**Authors:** Giang V. Pham, Brittany R. L. Duff

**Affiliations:** 1 Institute of Communications Research, University of Illinois at Urbana-Champaign, Urbana, Illinois, United States of America; 2 Sandage Department of Advertising, Beckman Institute for Advanced Science and Technology, University of Illinois at Urbana-Champaign, Urbana, Illinois, United States of America; University of Albany, State University of New York, UNITED STATES

## Abstract

Flow (state of intense focus) during media use has been largely considered a desirable experience, with technologies developed to maximize the chance of encountering flow in computer-mediated environments. However, the total absorption of attention due to flow could be problematic in contexts where the user has multiple predetermined goals, and engaging with the flow-inducing media could cost them resources that may be otherwise devoted to other goals. When flow imposes a cost on the user’s goal performance, it may also reduce their post-experience gratification with the flow-inducing media. The present study proposes a novel theoretical framework to begin understanding the potential cost of flow in media use with supporting evidence from two survey and vignette studies (*N* = 235 and *N* = 245). Its findings will extend human-computer interaction research by highlighting the double-edged impact that flow might have on media users’ larger goal performance and downstream well-being.

## Introduction

Every day, people around the world spend about eight hours with media [1]. In the U.S, the average adult uses their smartphone for four hours daily [2], and 48% of 18- to 29-year-olds state that they are “almost constantly” online [3]. There were an estimated 2.5 billion video gamers–nearly one-third of the world’s population–in 2020, with video game engagement reaching an all-time high during the COVID-19 pandemic [4]. Media use can offer emotional, cognitive, and social benefits [5, 6], however, it can also be problematic to users’ psychological wellbeing. For example, video-gaming has been found to distort sleeping among young people [7]; binge-watching has been shown to disrupt viewers’ daily task performance [8]; the Internet in general has been blamed for making people spend countless hours browsing aimlessly [9]. As media usage continues to reach new heights, it is critical to understand factors that help differentiate between media use that is positive or gratifying from media use that is problematic, disruptive, and causes people to ignore obligations due to the over-engagement.

The current study aims to extend this knowledge by looking at a potential underlying factor in both positive and problematic media use: flow. Flow experiences in media use and the positive benefits that they engender are well-known, however, with the positives there is also the potential for a negative cost that flow might impose on the user’s additional goal performance and downstream gratification. Flow [10] describes a state of absolute focus in an activity. A large body of research has shown that flow allows people to optimize and achieve enhancement effects within the activity where it occurs (e.g., [11, 12]). The present research, however, aims to investigate the downstream impact of flow in a context specifically designed to help facilitate flow experiences for users–media content (e.g., [13, 14]). Media users often have multiple ongoing goals in addition to just the pure engagement with the content and an intense concentration within one goal or activity can impede the performance of other goals–either concurrently or further down the line (e.g., [15]). In other words, we are examining the way flow, due to its extreme characteristics, can facilitate performing in one activity (the activity where it occurs) at the potential cost of the other. This could lead to unfavorable performance on other goal activities and retrospective gratification that challenge the assumption of flow as a purely positive state to achieve.

Consider a scenario in which a person plans to have thirty minutes of screen time before working on other tasks (e.g., doing chores/homework) for the evening. For their screen time, the person chooses media that matches their interest and ability and subsequently experiences flow (e.g., in playing a videogame or watching a show). The complete absorption experienced as part of flow may help the person achieve and enjoy the flow-inducing activity (gaming or watching) while doing it, however, it may also inadvertently divert their resources- including time and cognitive effort- away from their other predetermined goals (doing chores/homework). Thus, instead of the 30 minutes, they may spend two hours, and not be able to perform their other goal-directed activities as they had previously planned. As such, looking at the holistic experience, flow may not have been a desirable experience because it impeded the performance of the goals outside of the flow-inducing activity for the user. Acknowledging the current undesirable goal outcomes, the user’s retrospective gratification could be impacted such that they might experience regret (as opposed to pleasure) and potentially devalue the overall experience.

The present paper will review the experience of flow during media use and argue for its potential problematic aspects. It will then articulate a conceptual framework that highlights goal context as a potential determinant of flow’s outcomes: when using the flow-inducing media is the user’s only goal, flow can enhance goal achievement and gratification; however, when that media use is only one of the user’s multiple goals that are held concurrently or sequentially, the complete absorption caused by flow might disrupt goal performance and the gratification stemming from media use for the user. To distinguish this possibly problematic effect of flow from the well-studied positives of flow, a novel concept, *flow cost*, is proposed to describe the negative downstream influence of flow pertaining to overall goal performance and post-experience gratification. Preliminary empirical evidence of flow cost from two studies in which participants reported their everyday life experience with media flow and took part in a role-playing vignette will be presented.

The present research promises to make several contributions to media and human-computer interaction studies. First, by highlighting flow as a source of both positive but also potentially negative outcomes, it sheds light on the way flow underlies the process through which media can both facilitate and disrupt people’s day-to-day goal performance and gratification. Second, the conceptualization of flow cost should open new avenues of research which shift attention away from looking only at positives of flow within the media use to the boundary conditions in which flow may lead to both desirable and undesirable outcomes. Finally, outcomes from this research will deliver implications for developing technology that optimizes flow benefits (as opposed to flow cost) for media users, thereby promoting media utility and digital well-being.

### Flow experiences in media use

Flow [10] describes a psychological state in which an individual is devoting their full attention to an activity. During flow, people are so intensely focused on what they are doing that they are not directing attentional resources to any task-irrelevant activities, including self-referential thinking [16]. Flow was discovered from Csikszentmihalyi’s effort to understand what underlies artists’ peak performances; he found that artists feel their best and perform their best when they are ‘in the zone’- where the self and the surroundings vanish, the concept of time disappears, and every action leads seamlessly, fluidly to the next [17]. Over time, flow has been shown to be a universal experience that underlies people’s enjoyment and peak performance in various kinds of activities (see review in [18]).

Investigations into the psychophysiology of flow suggest that during the state, there is an upregulation of bodily functions necessary for performing the flow-inducing activity and a downregulation of functions that are irrelevant to the fulfillment of the flow-inducing activity [16, 19]. In particular, activation in neural networks related to higher-order attentional processing were observed during flow, indicating high attentional effort [20, 21]. At the same time, reduced activity in medial prefrontal areas and the default mode network were observed, suggesting an absence of self-referential thoughts and self-awareness during flow [22]. This explains why flow evidently demands high attentional effort but has been subjectively reported as “effortless”: for effort to be felt, people need to perform introspection, which seems to be abandoned during people’s complete focus within the flow-inducing activity. Weber and colleagues [23] theorized a synchronization process between attentional networks and reward networks during flow gives rise to the feeling of intrinsic enjoyment (see also [20]).

Flow has been employed to explain media enjoyment. Based on the uses and gratifications theory, which posits that people actively seek out specific media to achieve gratification [24], Sherry [12] argued that media use provides an ideal context for experiencing flow and that flow provides the means for satisfying myriad user gratifications. Media are often purposely created to capture user attention; attending to media could consume cognitive functioning such as information processing (e.g., during reading or watching) and/or problem solving (during playing video games) that activates a state of intense focus. For example, flow in reading has been found to be a common experience, as information–even novel storylines–can be complex and require efforts beyond reading literacy such as the ability to comprehend logical, historical, or cultural contexts to be processed [25]. When it comes to media, users can often select what best matches their ability and interest, allowing a balance between activity challenge (i.e., the complexity of the medium or mediated information) and skill (i.e., the user’s ability to control the medium or process the information) that is posited to be the necessary condition for flow [11, 25]. This is most applicable to video gaming wherein activity challenge and skill are clearly defined; however, it can also apply to other activities such as web browsing or video viewing because the activity itself does not arguably require extensive technical or literacy skills, but there is a little limit as to the complexity of the information. The intense concentration during flow allows media users to temporarily escape their personal concerns and excel in what they are doing, thereby achieving both hedonic (e.g., emotional pleasure) and non-hedonic gratification (e.g., competence; [6]).

Flow has been observed with various media activities: watching television [26], using social media [21], playing video games [20, 27], web browsing [28, 29], online shopping [30], etc. Researchers have argued that flow in media use (called media flow) share common characteristics with flow in other activities, including an intense focus, a merging of action and awareness, a sense of control, a loss of self-reflective consciousness, a distortion of temporal experience, and a feeling of intrinsic rewards [12, 27, 28, 30].

Flow might be becoming closer to the default way to experience media nowadays as users can easily access, control, and personalize media to match their own interests and skills. Most forms of media have been integrated with the technology purposely designed to maximize the attention and time users spend on them (e.g., see design principles for flow in [13, 14, 27]). For example, the infinite scroll on social media was created to make users endlessly browse through content and stay hooked [31] and the recommendation algorithm on Youtube was programmed to find the most relevant videos to each viewer’s interests and keep them watching [32]. There is also depth and breadth to the novel, interesting content readily available–from professionally produced to user-generated (e.g., there is more than 500 hours of video uploaded to YouTube every minute; [33])—making it possible for users to find things that appropriately match their interest and ability. In addition, the lack of boundaries in today’s computer-mediated environments enables users to seamlessly transition between content (e.g., from one video to another) and platforms (e.g, from social media to the web), thus, more likely to stay focused [9]. Thus, it is posited that the media today makes it much more likely for users to experience flow than in the past. Because these flow states may be easy to fall into, it may occur without user intention and therefore the state itself may disrupt the intended performance of other goals.

### Flow’s problematic aspects

Because flow was a concept developed out of an attempt to understand what makes people feel and perform their best [17], it has mainly been associated with positive, life-enriching implications. In addition, flow has mostly been studied with activities that are deemed meaningful such as art creation and performance, sports, meditation, etc. Even within the context of media use, engaging with media has been assumed to be the user’s desired end state without an investigation into when such engagement might not always welcome or preferred. When placed into the daily life context, flow’s extreme characteristics apparently have the potential to be problematic.

Flow has been shown to come with a reduced activity of the medial prefrontal cortex, an area of the brain best known for self-monitoring [19, 22, 34]. This reduction of monitoring can potentially lead to a lack of thinking about the compatibility between one’s current activity and one’s goals and obligations. For example, during flow, people won’t likely be able to recognize that spending hours playing video games could result in a conflict with their other goals–both short-term (e.g., writing, exercising) and long-term (e.g., being productive, maintaining social relationships). In addition, the potential production of pleasure-inducing neurochemicals such as dopamine during flow [19, 34] could make people subconsciously engage with the flow-inducing activity despite having other obligations. The distortion of time perception during flow can also be problematic given that people cannot always spend as much time as they wish on one activity without hindering other activities or goals. Thus, despite the enhancement effects within the flow-inducing activity, flow might come with significant trade-offs for people’s goal performance.

There exists evidence for the negative implications of flow experience during media use. Surveys have shown that flow is associated with longer gaming duration, less sleep, and higher regret experiences among adolescents [7, 35]. People who frequently experience media flow were found to be more likely to engage in problematic Internet use and videogame addiction [29, 36, 37]. Immersion in watching television (binge-watching) has been found to trigger goal conflict and feelings of guilt [8]. When smartphone users gained access to real-time reports about their time spent on the phone, many reported feeling “shocked” and “ashamed” at their own screen time usage [38], implying a lack of awareness while using smartphones that could be evidence of flow. Thus, there are contexts in which flow in media use is harmful to users’ wellbeing. However, there is not a theoretical foundation that helps explain the process through which flow as a pleasurable experience could lead to negative outcome; this motivates our theorization of potential flow cost in media use.

### Flow cost in media use

#### Flow cost on goal performance

To understand how flow might lead to negative outcomes, it is necessary to recognize that users can have different individual goals while engaging with media. A goal is defined as “a cognitive representation of a desired endpoint that impacts evaluations, emotions and behavior” [39]. A person can pursue one goal at a time (single-goal pursuit) or several goals simultaneously (multiple-goal pursuit). In fact, multiple-goal pursuit is prevalent in everyday life and people often have to allocate their limited time and attention resources among multiple activities [40, 41]. Using media is a usually a goal-directed behavior, with users actively seeking out media that meet their needs [24].

One of the fundamental obstacles of successful goal performance is failing to get started [40, 41]. To take action, people have to first remember their goal intention including what to do and when to do it–an act captured by prospective memory [42]. At its core, prospective memory is a process of information retrieval: people must allocate cognitive resources to obtaining information regarding their goal intention that has been stored in cognitive systems. Nowadays, media users also rely on means other than their own memory such as smartphone or laptop reminders to help them manage their activities. For these external cues to be effective, however, users need to direct their attentional resources to the cues in the first place.

Research has long shown that humans have a limited pool of cognitive and attentional resources at any given time (e.g., [43, 44]). When a person performs an activity, a part of their resource pool will be devoted to this activity. Depending on the requirements of the activity there could be few resources available in their pool to perform other activities simultaneously. Because a defining feature of flow is an absolute focus and it continuously demands a person to perform at their highest capability to maintain the skill-challenge balance [10, 11], when people are in flow, their cognitive and attentional resources would be strictly allocated to the flow-inducing activity. People might not yet reach the cap of their limited resource pool (because they are still able to carry on the activity), but they should narrowly focus their cognitive resources on the flow-inducing activity; this narrow focus is evidenced by the decreased activity in the medial prefrontal cortex [19, 22, 34].

If the flow-inducing activity is people’s only goal, flow allows them to become deeply engrossed in the activity and excel at it, as previous research has demonstrated [12, 20, 21, 23, 27]. However, if people are holding other predetermined goals, the full absorption of cognitive resources due to flow might prevent them from directing resources to retrieving information or attending to external cues regarding their goal intention. The distortion of temporal perception, a crucial component of flow, also results from the full absorption of resources during flow. Because the capacity for experiencing time is decreased by the requirement of resources for experiencing flow, people no longer have the conscious awareness of time passing by and would later recall a shorter duration of time spent on the flow-inducing activity [17, 18]. This distortion of time perception can make people truly unaware of how long they have been in the flow-inducing activity and thus unable to stop despite having spent the intended amount of time on it.

The sustainably high level of cognitive effort required for maintaining the experience of flow also makes it cognitively demanding, even though it is often subjectively reported as being effortless [16, 22]. Because sustained attention has been largely shown to be able to drain cognitive resources and lead to mental fatigue [7, 45–47], flow can potentially deplete cognitive resources for those experiencing it. Given that people have a limited capacity of both cognitive resources and time (24 hours a day), the more resources they devote to the flow-inducing activity, the fewer resources they would have available for performing goals outside of the flow-inducing activity. Thus, flow in one activity can result in a reduction of resources (time and effort) for other activities when people have multiple predetermined goals in a resource-limited context.

For example, a person originally plans to play a videogame for thirty minutes and then do homework, but, while playing, they experience a flow state that makes them unaware of how long they have been playing and unable to recall their intention to do homework or to pay attention to external reminders. Therefore, the person might now have less time and effort available for doing homework than originally intended, which could result in unsatisfactory homework performance, or take time and effort from other activities (e.g., exercising) to make up for the time lost in playing. In either case, flow has not facilitated but rather disrupted the optimal performance of goals outside of the media use.

The concept of *flow cost* is proposed to acknowledge the existence of negative downstream outcomes of flow and to differentiate flow’s potential problematic aspects from its well-known benefits. The first research question regarding flow cost on goal performance is:

*RQ1*. *Under the pursuit of multiple goals in a resource-limited context, can flow in media use impose a cost on the user’s performance of goals outside of the flow-inducing activity?*

#### Flow cost on gratification

Gratification comprises not only concurrent but also retrospective satisfaction regarding an experience [48]. Gratification in media use is often assessed by the extent to which people experience emotional pleasure from using the media (e.g., the level of enjoyment felt from watching a movie). Media scholars [12, 20, 21, 23] have argued that flow experiences during media use facilitate pleasure because they allow users to fully immerse in the media and dismiss any internal (e.g., one’s personal concerns) or external factors (e.g., distractors or requests) that could induce negative affect. However, if we make a distinction between concurrent and retrospective gratification, there might be occasions in which flow facilitates pleasure in the moment but undermines it afterward as users re-evaluate their course of action. Specifically, users might experience pleasure while using media/experiencing flow, but once they stop the media use and realize that flow has harmed their goal performance, this feeling of pleasure might be reduced.

People tend to value things that are relevant and devalue things that are irrelevant to their current goal; the phenomena known as ‘valuation’ and ‘devaluation’ effects [49, 50]. Essentially, the valuation effect proposes that an active goal increases the attractiveness of the things that are instrumental for its satisfaction (e.g., a hungry person tends to value bread above water, but a thirsty person would value water above bread). When a user is in flow, the goal within the flow-inducing activity (e.g., to learn, to get entertained, to be challenged) is focal to them. Therefore, the flow state, which enhances the achievement of this goal by allowing users to be deeply engrossed in the activity, is positively valued at that time. This valuation effect is expressed through a high level of pleasure frequently observed through self-reports and physiological measures during flow [19, 20, 21, 26, 27, 30, 34]. There should be no differences in concurrent gratification between goal contexts (single vs. multiple predetermined goals) because during this time, people are still completely focused on the flow-inducing activity and are not likely able to do any self-referential thinking that enables them to appraise their goal performance status.

The differences might appear in retrospective gratification, after people have stopped their flow-inducing media use. When flow contributes to the accomplishment of a goal and doesn’t impact the performance of other goals (e.g., when someone can devote their entire evening to playing video games), the positive valuation of the flow experience should continue such that people would still experience a sense of satisfaction after flow has ended. This may be why media flow has been shown to result in retrospective enjoyment, it has been studied largely in contexts where using media is the user’s only goal (e.g., [12, 27, 28]). However, in a multiple-goal context where flow has imposed a cost on the user’s goal performance (e.g., when someone has missed or delayed their homework due to the experience of flow in playing video games), the user will be likely to recognize how their goals have been overshadowed due to their engagement with the media when flow has ended. At this point, the goals outside of the flow-inducing activity (studying) have once again become active and focal for the user. The absolute focus in the media activity (flow)- instead of being a facilitator- is likely to be recognized as a disruptor to their currently active goal. Therefore, it is posited that the devaluation effect will take place, such that the user will be less likely to feel a retrospective satisfaction about the flow-inducing media than they would in a single-goal context, nor feel the continuation of the pleasure carried over from flow.

In the example above, the person who has realized that their playing videogames has disrupted their goal to study would be less likely to feel satisfied afterward than the person who didn’t have their subsequent goal performance impacted, even though both of them felt pleasure while playing the videogame. Because the ultimate outcome (i.e., the delay of homework) is not their intended nor preferred outcome, they might also experience regret. This experience is similar to people’s feeling of guilt when failing to comply with their planned schedule or when committing impulsive behavior (e.g., [8, 48, 51]). In these situations, flow might have facilitated concurrent gratification for the user during media use, but this gratification is likely to be undermined once the user has resumed introspection and recognized the unfavorable goal outcome caused by the flow experience. The following research question regarding the cost of media flow on user gratification is proposed:

*RQ2*. *When flow in media use has imposed a cost on the user’s goal performance, can it also impose a cost on their retrospective feeling of gratification from the flow-inducing media?*

### Preliminary evidence of flow cost

To provide some initial evidence of this process possibly occurring with everyday media use, we conducted two online studies, each consisting of a vignette experiment and a survey. Vignette or scenario-based role-playing experiments are experiments in which participants are assigned to play a defined role, and through this role, react to scripted information presenting specific factors of interest in a study [52]. This method has proven valuable for yielding insights about complex, difficult to operationalize, or sensitive attitudes and behaviors that often change in response to contextual factors (e.g., consumer ethics; political preferences; [53, 54]).

While flow has been well-studied, the induction of flow in lab experiments requires a validation of the flow-inducing task at different levels and thus requires a narrowing of the specific type of task and/or participant. Because we are interested in exploring whether flow cost is something that would occur across people and media use activities, simulating real-life scenarios of media use that randomly vary in goal context will allow for preliminary insights into this phenomenon. A 2 within-subject (goal context: single vs. multiple) by 5 between-subject (activity: watching television, playing video games, browsing social media, online shopping, editing videos) study design was employed.

#### Scenario vignettes

To ensure that participants’ responses were not particular to a specific media use activity, we created vignettes that describe the experience of media flow in five different activities. Each vignette describes a scenario of media use in which a person has no intended goals or obligations that could later concern their initial media use (single goal context- no flow cost) or have another goal they intended to complete further down the line (multiple goal context- flow cost imposed) within a time-limited context. The experience of flow, especially the language used, is derived from the description and characteristics of flow identified in previous research [12, 30]. The flow cost on goal performance is conveyed through the description of a predetermined goal (not media related) that is eventually dismissed or delayed due to the user’s experience of flow during media use. More specifically, these scenarios describe that the user has time available for using media at the present, but they are also planning to do another activity (e.g., going out, meeting a friend, working) some time from the present, which they eventually delay or dismiss because of their complete engagement with the media. The initial scenarios were developed with informal feedback from several undergraduate students and a pilot study was conducted to validate them. Aside from the goal context, all elements are kept identical between the paired vignettes in each media use activity. A sample pair of vignettes are shown in Table 1.

**Table 1:** Example of the scenario vignettes (activity: Watching TV).

Single goal context (No flow cost)	Multiple goal context (Flow cost imposed)
Goal context It’s weekend and you finally get to watch the TV show you have been curious about. You have completed your chores, had some snacks ready, and put your phone in silent mode so you can enjoy watching.	Goal context It’s weekend and you finally get to watch the TV show you have been curious about. You plan to watch for about an hour or two then go out for a run and meet up with some friends since it’s nice outside.
Flow experience Soon after the show starts, you become totally engrossed in watching and you are no longer aware of time and your surroundings. You watch one episode after another almost automatically. You are so entirely focused on learning what’s going on that you are not thinking of anything that is not related to the show. You don’t check your phone even once.	Flow experience Soon after the show starts, you become totally engrossed in watching and you are no longer aware of time and your surroundings. You watch one episode after another almost automatically. You are so entirely focused on learning what’s going on that you are not thinking of anything that is not related to the show. You don’t check your phone even once.
Goal performance results Hours have gone by until you realize that you have been watching for a long time and finished numerous episodes. You have been so engaged with watching the show that you didn’t notice how long or how much you have watched.	Goal performance results Hours have gone by until you realize that you have been watching for a long time and finished numerous episodes. It’s getting late, and you have spent most of the day on your couch instead of going running and seeing your friends as planned.

#### Study procedure

Participants were informed that the current study aimed to understand media use in everyday life. Participants entering the online study site were randomly assigned to one of the study conditions. They were first asked to read and imagine themselves in a randomized scenario wherein media flow does or does not result in a cost on goal performance. After that, they indicated the extent to which they could relate to the scenario and answered questions that assess their perceived feeling of pleasure and regret during and after the engagement with the media in the scenario. Then, they were assigned to read another semi-randomized scenario for the other condition: if they first read a multiple goal scenario, they now read a single goal scenario and followed the same procedure. The study was set up so that participants didn’t read two scenarios describing the same activity. To validate participants’ ability to relate to the study scenarios, a narrative description of flow experiences adapted from previous research [30] was provided, followed by the question “Have you ever experienced flow while using media?” (Yes/No). Participants who responded “No” (*N* = 5) were skipped to the end of the study and were excluded from the study sample, while participants who responded “Yes” proceeded with questions about their personal experiences with media flow. Finally, participants reported their demographic information and left the study site.

#### Ethics statement

This study’s protocol was approved by the Institutional Review Board (IRB) at University of Illinois from where the research was conducted. We obtained electronic consent from participants and a waiver of documentation of consent.

#### Measures

We measured participants’ *ability to relate to the scenario* using three items adapted and modified from [53]: “How difficult or easy is it for you to imagine yourself in this scenario?”, “How vividly could you imagine the scenario that was described?”, “How relatable are the feelings in this scenario to you?”, Cronbach’s *α* = .928.

After reading each scenario, participants were asked to separately indicate how they think they would feel during and after their engagement with the media has ended. The *perceived feeling of pleasure* during and post-media use were assessed by three items adapted from previous research [55]: pleased, gratified, contented, with Cronbach’s *α* of .873 and .788, respectively. The *perceived feeling of regret* during and post-media use were assessed by three items adapted from previous research [56]: upset, disappointed, regretful, with Cronbach’s *α* of .848 and .823, respectively.

The *retrospective gratification from media use* was assessed by four self-report items adapted and modified from [57] asking participants how they felt about their engagement with the media in the scenario: “It was rewarding,” “It was satisfying,” “My time was well-spent,” and “It was a waste of my time (reverse coded)”; Cronbach’s *α* = .819.

Participants’ *frequency of experiencing media flow* was assessed by two 7-point anchors (1 = Very rarely, 7 = Very frequently; 1 = Almost never, 7 = Very often) to the question “How often do you experience flow while using media?” following a narrative description of flow adapted and modified from Novak and colleagues [30]; Cronbach’s *α* = .917. This method has been proven an effective way to gain insights into people’s personal encounters with flow.

Participants’ *frequency of experiencing flow cost on goal performance* was assessed by the social media self-control failure (SMSCF)-scale adapted from Du and colleagues [58]: “How often, because of experiencing flow while using media, do you continue to use media even though your media use at that time: (1) conflicts with your other goals (for example: doing things for study/work/family)?, (2) makes you use your time less efficiently?, (3) makes you miss or delay other things you want or need to do?”; Cronbach’s *α* = .895. Only participants who indicated that they have experienced flow while using media responded to this question. The SMSCF scale was established to assess how often social media users give in to social media temptations, resulting in undesirable goal outcomes. This is similar to the experience of flow cost in that both phenomena describe how users’ goals are dismissed because of their over engagement with the media. Thus, by replacing social media use with the experience of media flow, the scale provides a means for accessing the how often users let media flow affects their goal performance.

Participants were also asked to list two to five media use activities that they have personally experienced flow with. Separately, they listed two to five activities they have personally experienced flow with that made them miss or delay something that they intended to do. A list of activity categories was generated by two graduate student coders, with a general category created for each activity reported (e.g., playing Minecraft and playing Candy Crush are coded as playing video/mobile games; watching Friends is coded as watching TV/movies). If a specific media platform was mentioned, the activity was coded as the general activity associated with that media platform (e.g., “Facebook” coded as “using social media”, “Youtube” coded as “watching online videos”). Each listed activity was coded as “1” if it belongs in a category and coded as “0” if it doesn’t belong in that category. The ratio of the total count of a category to the total number of activities listed is used to determine their percentage in the summary table (Table 2). A subset of data (20%) was selected for the coders to code independently, and an intercoder reliability (Krippendorf’s *alpha*) of .91 was found, indicating a high level of coder agreement.

**Table 2:** Summary of activities reported for flow and flow cost experiences in media use.

Media activity	Pilot study (*N* = 235)		Main study (*N* = 245)	
	Flow	Flow cost	Flow	Flow cost
Using social media	29.4%	27.0%	12.7%	13.3%
Watching TV/movies	20.3%	22.6%	18.0%	23%
Watching online videos	14.5%	14.2%	8.5%	7.2%
Playing video/mobile games	8.0%	7.4%	12.9%	9.4%
Online shopping	6.1%	5.0%	1.8%	2.9%
Communication (e.g., texting)	4.8%	4.2%	5.6%	4.4%
Listening to music/radio/podcasts	3.6%	5.0%	8.9%	5.9%
Web-browsing	3.4%	5.3%	7.2%	6.2%
Reading (e.g., novels, blogs)	2.8%	1.5%	7.1%	5.6%
Designing (e.g., graphics, videos)	2.5%	3.3%	2.8%	2.9%
Studying or working	2.1%	2.1%	5.5%	7.4%
Watching or reading news	1.8%	1.8%	6.7%	6.2%
Watching sports	0.4%	0.3%	1.7%	5.3%
Watching advertisements	0.3%	0.3%	0.6%	0.3%

### Pilot study

A pilot study was conducted to validate the study material and procedure. 235 undergraduate students (*N* = 235; 59.1% females; *M*_*age*_ = 19.82; *SD*_*age*_ = 1.22) at a large Midwestern university participated in the study in exchange for course credits. The race of participants are: White (68.5%), Asian (20%), American Indian or Alaska Native (5.1%), Native Hawaiian or Other Pacific Islander (3.4%), and Black or African American (3.0%).

### Pilot study—Findings

We first report participants’ personal experience with flow and flow cost on goal performance in media use. Participants reported an average frequency of *M* = 4.33 (*SD* = 1.18) of experiencing flow while using media, and an average frequency of *M* = 4.02 (*SD* = 1.22) of experiencing media flow’s cost on goal performance. Given the 1–7 frequency scale, these numbers suggest that participants feel that they have experienced both flow and flow cost more than sometimes, but they are not things that always occur. Table 2 shows a detailed summary of the media use activities in which participants reported having experienced flow (cost). However, because we had participants indicate their personal experiences only after the scenario vignettes, we were concerned whether their response was primed by what was described in the scenarios. Hence, there is a need to examine participants’ personal experience with flow without the potential influence of the scenarios.

To understand how people might relate to the experience of flow cost, we examined participants’ response to the scenario vignettes. There was no significant impact of the specific media activity on the dependent variables, including ability to relate to the scenario (*F*(4, 230) = .35, *p* > .1), perceived feeling of pleasure during both media use (*F*(4, 230) = .89, *p* > .1) and post-media use (*F*(4, 230) = 1.42, *p* > .1), perceived feeling of regret both during media use (*F*(4, 230) = 1.58, *p* > .1) and post-media use (*F*(4, 230) = 1.23, *p* > .1), and perceived gratification from media use (*F*(4, 230) = .65, *p* > .1); hence, we combined analyses across media activities and conducted paired t-tests to compare the dependent variables in the flow cost (yes vs. no) conditions.

Overall, participants indicated that they could (significantly above the scale midpoint) relate to both multiple goal scenarios (*M* = 4.49, *SD* = 1.61; *t*(1, 234) = 4.63, *p* < .001) and single goal scenarios (*M* = 5.17, *SD* = 1.38; *t*(1, 234) = 12.95, *p* < .001). Table 3 shows the study’s descriptive statistics. Participants’ indicated personal experience, along with their ability to relate to the scenarios, suggest our study’s approach does tap into people’s subjective experiences with flow in media use. More importantly, they provide support for the possibility of flow cost on goal performance under the pursuit of multiple goals, which addresses *RQ1*.

**Table 3:** Descriptive statistics in vignette studies.

	Pilot study (*N* = 235)		Main study (*N* = 245)	
	Single goal context (No flow cost)	Multiple goal context (Flow cost)	Single goal context (No flow cost)	Multiple goal context (Flow cost)
Ability to relate to the scenario	5.17 (1.38)	4.49 (1.61)	5.49 (1.26)	5.40 (1.33)
During media use				
Perceived feeling of pleasure	5.71 (1.04)	5.48 (1.14)	5.48 (1.43)	5.37 (1.25)
Perceived feeling of regret	2.26 (1.13)	2.53 (1.35)	3.18 (2.08)	3.19 (2.05)
Post-media use				
Perceived feeling of pleasure	4.56 (1.46)	3.45 (1.65)	5.19 (1.39)	3.99 (1.89)
Perceived feeling of regret	3.50 (1.73)	4.72 (1.73)	3.51 (2.02)	4.70 (1.96)
Perceived retrospective gratification from media use	4.23 (1.32)	3.49 (1.22)	4.81 (1.22)	3.87 (1.54)

*Note*. Scale of 1 to 7 with 1 = Strongly disagree, 7 = Strongly agree.

Participants reported a significantly higher perceived feeling of pleasure during media use for single goal scenarios (*M* = 5.71, *SD* = 1.04) than for multiple goal scenarios (*M* = 5.48, *SD* = 1.14), *t*(1, 234) = 3.20, *p* = .002, Cohen’s *d* = .21. Similarly, participants reported a significantly lower perceived feeling of regret during media use for single goal scenarios (*M* = 2.26, *SD* = 1.13) than for multiple goal scenarios; *M* = 2.53, *SD* = 1.35), *t*(1, 234) = −3.22, *p* = .001, Cohen’s *d* = −.21. Hence, the pilot study failed to find support for the position that people would perceive similar levels of pleasure while experiencing media flow despite the goal context. While it is possible that people would perceive a higher feeling of pleasure from media use when they don’t initially have other goals planned, this result may also be an artifact of how we assessed participants’ perceived feelings of pleasure and regret. Specifically, participants were asked to indicate how they might feel during the media use only after they have read the entire vignette. This means that for multiple goal scenarios, participants already know that their engagement with media use will lead to a disruption of other goal performance when responding to the measure. Thus, the assessment of perceived pleasure and regret during media use should be changed to eliminate the potential impact of this knowledge.

Participants reported a significantly higher perceived feeling of pleasure post-media use for single goal scenarios (*M* = 4.56, *SD* = 1.46) than for multiple goal scenarios (*M* = 3.45, *SD* = 1.65); *t*(1, 234) = 8.36, *p* < .001, Cohen’s *d* = .55. Similarly, they reported a significantly lower perceived feeling of regret post-media use for single goal scenarios (*M* = 3.50, *SD* = 1.73) than for multiple goal scenarios (*M* = 4.72, *SD* = 1.73); *t*(1, 234) = −8.93, *p* < .001, Cohen’s *d* = −.58. The same pattern of results was found for perceived retrospective gratification from media use: participants perceived a significantly higher gratification for single goal scenarios (*M* = 4.23, *SD* = 1.32) than for multiple goal scenarios (*M* = 3.49, *SD* = 1.22); *t*(1, 234) = 3.49, *p* < .001, Cohen’s *d* = .46. These findings suggest that participants tend to retrospectively appraise their engagement with the flow-inducing media as less pleasurable, more regretful, and generally less gratifying in situations that media flow has imposed a cost on their goal performance compared to when it does not. Thus, *RQ2* is affirmatively addressed.

### Main study—Method

The main study was conducted to replicate the pilot study with two changes in the study procedure. First, participants completed the survey section that assessed their personal experiences with flow and possible flow costs in media use before completing the vignette study. This procedure helps prevent the potential priming effects of the scenario vignettes and enables participants who felt that they have never experienced media flow to be dropped (*N* = 9) earlier in the study. Second, in the vignette study, participants first read the beginning of the scenario that describes the goal context and the flow experience and then indicated what they might feel during that media use. After everyone read the same scenario of experiencing media flow, they then read the ending of the scenario describing the results of their overall goal performance and indicated what they might feel post-media use (see vignette examples in Table 1). This procedure helps ensure that participants’ perceived feelings during media use were not influenced by the outcome of the scenario and more closely mimics the actual process (i.e., users being unaware of the potential downstream consequences of flow during the time experiencing it).

The final sample includes 245 Amazon Mechanical Turk participants (*N* = 245; 42.4% females; *M*_*age*_ = 32.06, *SD*_*age*_ = 9.67); the majority identified as White (53.5%), followed by Asian (34.7%), American Indian or Alaska Native (6.9%), Black or African American (4.1%), and Native Hawaiian or Other Pacific Islander (0.8%).

#### Measures

The main study employed measures from the pilot study; each had good reliability with the following Cronbach’s α: Frequency of experiencing media flow (.828), Frequency of experiencing flow cost on goal performance (.898), Ability to relate to the scenario (.873), Perceived feeling of pleasure during (.888) and post-media use (.884), Perceived feeling of regret during (.974) and post-media use (.870), Perceived retrospective gratification from media use (.807). The intercoder reliability (Krippendorf’s *alpha*) for the coding of activities in which participants reported having experienced media flow (and its diversion effect) is .93.

### Main study—Findings

Participants reported an average frequency of 5.33 (*M* = 5.33, *SD* = 1.35) of experiencing media flow and an average frequency of 4.65 (*M* = 4.65, *SD* = 1.52) of experiencing a flow cost on goal performance, suggesting that they feel they have experienced both flow and costs from flow somewhat often. The most popular activities in which participants reported having experienced flow (cost) are: watching TV/movies, using social media, playing video/mobile games, watching online videos, and web-browsing (see Table 2). Participants indicated that they could (significantly above the midpoint) relate to single goal scenarios (*M* = 5.49, *SD* = 1.26; *t*(1, 244) = 18.55, *p* < .001) and multiple goal scenarios (*M* = 5.40, *SD* = 1.33; *t*(1, 244) = 16.42, *p* < .001), providing affirmative support for *RQ1* (see Table 3).

Because there was no significant impact from differences in the media activities used in the vignettes, we combined the responses across specific media types and compared goal context (single vs. multiple) conditions. Participants self-reported similar levels of feelings of pleasure and feelings of regret during media use in these two conditions. Specifically, there was no difference in perceived feeling of pleasure during media use between single goal scenarios (*M* = 5.48, *SD* = 1.43) and multiple goal scenarios (*M* = 5.37, *SD* = 1.25); *t*(1, 244) = 1.47, *p* > .05. Likewise, there were no difference in the feeling of regret while imagining the flow-inducing media use between single goal scenarios (*M* = 3.18; *SD* = 1.25) and multiple goal scenarios (*M* = 3.19, *SD* = 2.05); *t*(1, 244) = .11, *p* > .05.

Post-media use, participants reported a significantly higher feeling of pleasure for single goal scenarios (*M* = 5.19, *SD* = 1.39) than for multiple goal scenarios (*M* = 3.99, *SD* = 1.89); *t*(1, 244) = 10.11, *p* < .001, Cohen’s *d* = .65. Similarly, participants reported a significantly lower perceived feeling of regret post-media use for single goal scenarios (*M* = 3.51, *SD* = 2.02) than for multiple goal scenarios (*M* = 4.70, *SD* = 1.96); *t*(1, 244) = −8.40, *p* < .001, Cohen’s *d* = −.54. A similar pattern of results was found for perceived gratification from media use, with participants reporting higher retrospective gratification for single goal scenarios (*M* = 4.81, *SD* = 1.22) than for multiple goal scenarios (*M* = 3.87, *SD* = 1.54); *t*(1, 244) = 8.59, *p* < .001, Cohen’s *d* = .55. Together, the results suggest that users tend to perceive feeling high gratification during media flow, however, after the flow state has ended, their sense of gratification might be diminished as they realize that their engagement with the flow-inducing media has imposed a cost on their subsequent goal performance, providing an affirmative answer to *RQ2*.

## Discussion

The enhancement effects of flow within the activity where it occurs have been widely recognized, with technologies developed to optimize the possibility of experiencing flow in computer-mediated environments (e.g., [13, 14, 31, 32]). However, the positives of flow states may come with a potential downside: flow may facilitate one goal at the cost of the others because people have limited capacity in terms of time and cognitive resources for everyday life activities. Arguing for the a broadening in how we think of the holistic outcomes of flow, we theorized and presented preliminary evidence for how the goal context in which people engage with media might determine when flow experiences could be problematic. Results from two exploratory studies provided initial support for our propositions regarding the possibility of flow costs on media users’ downstream goal performance and gratification.

Survey methods have been popular in studying flow (e.g., [7, 29, 30, 35–37]), because flow’s extreme characteristics make it likely for people to subjectively acknowledge and recall the experience. Participants in our study reported having encountered flow and flow cost often, especially while engaging with entertainment media such as TV/movies/online videos, social media, and video/mobile games. Experiences with flow (cost) in productive media use such as studying and designing were reported by a small portion of the sample. Although these findings are not generalizable to indicate media consumption trends, they provide insights into how prevalent flow and flow cost may be nowadays. People can become overly immersed in using entertainment media to the point that their other tasks and goals are ignored. This is in-line with previous reports that showed that the desire to use media is among the most difficult temptations to resist when conflicting with other tasks [59]; flow could underpin what makes media use such an irresistible temptation. Because content and platforms may be designed to induce flow, flow may occur more effortlessly in these contexts, with less intention to fall into the state than when one has to create the ideal scenario for its occurrence. This phenomenon may be exacerbated in today’s computer-mediated environments where media can be used to fulfill myriad goals and there is little boundary between different content and activities.

Even though this study did not provide direct evidence of flow cost, participants’ response to the scenarios are notable indicators of the potential way through which flow in media use could negatively impacts users’ overall goal performance and retrospective gratification. The samples were screened to only include people who have encountered flow in media use (a large portion of all who were recruited–more than 98% in the pilot study and more than 96% in the main study), so that they could reflect on their personal experience with flow. Participants’ ratings on their ability to relate to the scenario indicated that they found the scenarios realistic and familiar. Therefore, even though flow was not induced, participants felt that they could relate and had a recollection of what the experience is like. As evidence, they reported a high perceived feeling of pleasure during media use which is typical of flow.

In the flow cost scenarios, the experience of flow led to missing or delaying another goal (activity), which is our operationalization of flow cost on goal performance in this study. Even though we did not directly observe participants while they were experiencing a flow state, we believe that people generally have a sense of what flow is like and also what it means to have a planned goal disrupted by something (in this case, the complete focus in using media); therefore, what participants reported feeling here could be indicative of what they actually feel when encountering these situations. Because any given participant randomly saw two of the scenarios, the differences in their responses were most likely due to the differences in the goal context and the negative downstream impact of flow on subsequent goal performance within the scenario.

Results from the vignette studies implied that even though participants reported the perception of a high level of emotional gratification while using the flow-inducing media, in contexts where flow ultimately harms other goal performance, they eventually anticipated lowered retrospective media use gratification. The similarity in reporting feelings of pleasure and regret during media use in the two conditions (flow cost: yes vs. no) wasn’t found in the pilot study, however, after modifying the study procedure to eliminate effects of retrospective evaluation of the holistic scenario (rating flow based on what they knew about the overall outcome, not just the flow activity), it emerged in the main study. This finding is critical because it suggests that the acknowledegement of the undesirable goal outcomes only comes after a flow state has ended; these outcomes might affect how people appraise their overall experience.

Overall, our findings suggest: (1) there might be occasions in which flow in media use impedes the performance of goals outside of the flow-inducing media activity for users, and (2) when flow imposes a cost on goal performance, it might also result in a cost on retrospective gratification about the whole experience. This cost on gratification could feed into a cost on psychological wellbeing, because they might experience negative feelings such as regret and guilt from failing to comply with their goals and obligations, they might also experience discontentment from what they may perceive as a lack of control over their media use. These areas will need future work to test and disentangle them.

### Implications of flow cost

We believe that flow cost presents a topic worthy of study for human-computer interaction and media psychology scholars. A robust body of literature has shown that media use frequently diverts users’ attention and effort away from their daily activities, causing goal conflicts and emotional distress (e.g., [29, 35–37, 60]). The displacement theory, for example, posits that time spent using media displaces time spent on other important activities in one’s life [61]. The theorization of flow cost expands this position by adding insights into how displacement takes place during the flow experience.

Deficits in self-control have been believed to account for media overuse (e.g., [58, 60]). Even though there is still much work to be done to understanding how flow may affect cognitive resources within a given timeframe, as a sustained attentional state [16, 21, 22, 34], flow could drain the cognitive resources that would be needed for performing other activities in a resource-limited context. As such, flow cost might potentially offer an alternative explanation for how the engagement with media absorbs the resources that could be used toward exerting self-control, resulting in problematic media use and other goal failures. Unlike procrastination, which describes people’s intentional delay of an activity because they anticipate a lack of intrinsic rewards in that activity [29, 48], flow cost explains the delay of activities due to a lack of self-referential thinking experienced as part of flow, which occurs rather unintentionally. While the media user has largely been blamed for problematic media use (e.g., because of a lack of self-control; [8, 58]), the study of flow cost raises concerns regarding the responsibility of the media that are designed to more effortlessly induce flow. Research on flow costs could help people better understand the nature of underlying causes of problematic media use and thus better avoid undesirable consequences.

By demonstrating how media flow could be a double-edged sword for user gratification, the theorization of flow cost also extends the area of media and psychological well-being (e.g., [6, 48]). On one hand, flow can facilitate gratification and competence in media activity for users; on the other hand, when a complete engagement with media is not truly desired, flow can lead to downstream psychological conflict and negative affect (e.g., regret, guilt) as users re-examine their course of action. The priority or the value of the goal driving the flow-inducing activity versus the goal(s) that are harmed by flow might potentially predict the magnitude of flow cost. For example, a delay or missing of goals might be less significant to users if using media has fulfilled a more prioritized goal (e.g., learning or escapism) for them compared to what they have missed (e.g., doing chores). Future research on factors that can increase or mitigate flow cost will warrant meaningful insights into the moderators of the relationship between media use and users’ well-being.

Considering how prevalent media flow might be nowadays, the understanding of contexts in which encountering flow would be problematic for users (e.g., when they have several obligations for the day) could be extremely valuable. The investigation into human and design factors that help mitigate flow cost would inform the development of practical tools such as intelligent systems (e.g., time management apps, virtual assistants) that can detect and intervene in undesirable flow experiences. Given the increasing attention to technology that support people’s well-being (e.g., [62]), the knowledge of flow cost can deliver important implications for the design of product features (e.g., notifications, scheduling, blocking) that promote positive engagement with media while reducing negative aspects of it.

Flow cost might not be restricted to media use. Because resources for everyday life activities are limited, the complete focus on one activity may come with trade-offs for other activities. When flow takes place in activities that are meaningful or provide long-term benefits to those experiencing it (e.g., creating or performing arts, playing sports, working), the positives it brings could outweigh the negatives it causes. However, because using media–especially entertainment media–may frequently provide instant rather than long-term gratifications, the cost of flow experiences in media use could carry more weight than the cost of flow in other activities. Therefore, our conceptualization of flow cost broadens flow research by highlighting that flow is not always a constructive experience when considered broadly, rather, its outcome may be determined by the goal driving the flow-inducing activity in relation to other goals.

### Limitations and future research

Our study has several limitations. First, the use of the vignettes restricts our ability to make inferences about the mechanism underlying flow cost on goal performance and user gratification. We relied on participants’ ability to project their feelings and perceptions to hypothetical situations rather than examining their response while actually experiencing flow and flow cost; this could create room for confounding factors such as individual media use tendencies or ability to fictionalize oneself in a hypothetical situation. It also made it difficult to separate a participant’s own perceptions from their understanding of social norms (i.e., their belief on what is likely to happen). To validate the proposed theoretical framework, future research should examine the impact of media flow on goal performance and user gratification in a more controlled setting with an induction of media flow in single- vs. multiple-goal contexts.

Our study also failed to consider the potential roles of varying aspects of goal pursuit in the outcomes of flow. For example, the type or proximity of a goal might determine whether flow cost necessarily embodies a devaluation of media use. If people experience flow during a media activity driven by a long-term goal (e.g., learning), the missing or delay of a short-term goal (e.g., washing dishes) might not influence their gratification as much as it would otherwise do. Similarly, if media flow impedes the performance of a goal with distant consequences from the present (e.g. writing a paper that is due in two weeks), it might not impose as serious a cost as when it impedes the performance of a goal with immediate consequences (e.g. writing a paper that is due the next day). Thus, future efforts should be directed toward identifying different goal factors that may affect the experience of flow cost.

Another intriguing area for future search is to examine the interplay between flow cost and other factors responsible for the delay of tasks and goals due to media use such as self-control deficiencies and procrastination. Because the methods for overcoming problematic media use depend on its cause, it will be important to understand how media flow comes into play with other factors in facilitating problematic media behavior. In addition, examining the roles of dispositional factors that influence media choice and consumption such as impulsivity, sensation seeking, need for cognition, in explaining the occurrence and outcomes of flow cost also appears to be a promising endeavor for future research. Finally, the study of how flow cost could potentially impact users’ affective and behavioral responses to media content and communication efforts exposed to them during media flow would provide implications for improving the effectiveness of media placement.

Even though the present study is mainly theoretical and further research is needed to understand how flow may affect cognitive resources within a given timeframe, it has laid out a conceptual foundation for examining the way flow experiences in media use negatively impact factors critical to people’s well-being: goal performance and gratification. It has also presented preliminary evidence that suggests that flow cost could be a prevalent experience in everyday media use. We hope that researchers would find this line of research worthwhile and join us in trying to understand the potential for broader implications of flow states on wellbeing.
